# An Energy-Efficient Method for Human Activity Recognition with Segment-Level Change Detection and Deep Learning

**DOI:** 10.3390/s19173688

**Published:** 2019-08-25

**Authors:** Chi Yoon Jeong, Mooseop Kim

**Affiliations:** Human Enhancement & Assistive Technology Research Section, Artificial Intelligence Research Lab., Electronics Telecommunications Research Institute (ETRI), Daejeon 34129, Korea

**Keywords:** human activity recognition, fully convolutional network, segment-level change detection, energy-efficient method, deep learning

## Abstract

Human activity recognition (HAR), which is important in context awareness services, needs to occur continuously in daily life, owing to which an energy-efficient method is needed. However, because human activities have a longer cycle than HAR methods, which have analysis cycles of a few seconds, continuous classification of human activities using these methods is computationally and energy inefficient. Therefore, we propose segment-level change detection to identify activity change with very low computational complexity. Additionally, a fully convolutional network (FCN) with a high recognition rate is used to classify the activity only when activity change occurs. We compared the accuracy and energy consumption of the proposed method with that of a method based on a convolutional neural network (CNN) by using a public dataset on different embedded platforms. The experimental results showed that, although the recognition rate of the proposed FCN model is similar to that of the CNN model, the former requires only 10% of the network parameters of the CNN model. In addition, our experiments to measure the energy consumption on the embedded platforms showed that the proposed method uses as much as 6.5 times less energy than the CNN-based method when only HAR energy consumption is compared.

## 1. Introduction

The recognition of human activity plays a vital role in context awareness services such as daily lifelogging, surveillance, healthcare, and human-computer interaction. The worldwide availability of smartphones equipped with inertial measurement unit (IMU) sensors has increased the interest in the use of these devices for Human Activity Recognition (HAR) to monitor and track daily activities.

Previous research on HAR using smartphones mainly involved two approaches: methods based on handcrafted features and deep-learning-based methods. The former of these methods generally extracts features from the raw signals of IMU sensors and then classifies the activity by using either machine learning or statistical analysis methods. However, handcrafted features poorly generalize to other tasks because they are designed for specific tasks [1]. Moreover, the discriminative power of handcrafted features is usually low.

In recent years, deep neural networks (DNNs) have yielded satisfactory results in audio and visual recognition tasks because of their ability to systematically and automatically learn discriminative features from training samples [2,3]. Thus, many studies on HAR tasks using the superior power of DNNs have been reported [1,4,5,6,7,8,9,10,11]. Various DNNs such as convolutional neural networks (CNNs) [4,5,6,7,8], recurrent neural networks (RNNs) [9,10,11], and restricted Boltzmann machines (RBMs) [1] have been used to classify human activities and have shown impressive results in terms of their accuracy. However, the disadvantage of DNNs is their high computational costs to achieve high accuracy. This limitation would render them unsuitable for deployment on embedded devices, which would require the energy consumption of the deep-learning methods used for HAR to be considered. Especially, the need to frequently recharge monitoring devices can result in user dissatisfaction because a user activity must be monitored over an extended period to ensure the results are meaningful and because monitoring devices such as smartphones or embedded devices have limited battery and computing power.

An HAR system using deep learning comprises two major parts: segmentation and activity classification. The segmentation procedure preprocesses raw sensor signals to reduce the noise components before dividing the filtered sensor signals into smaller groups of signals using fixed-size windows. The subsequent activity classification process classifies human activity based on the information contained in these segments. The classification results for each segment can be fused at a later stage to increase the activity recognition rate.

Many methods have been proposed to reduce the energy consumption of the activity classification process as part of the overall system. HAR methods based on handcrafted features mainly reduce the energy consumption by lowering or varying the sampling rate of the inertial sensors [12,13]. In addition, several methods using shallow networks to reduce the energy consumption of activity recognition engines based on DNNs were proposed [14,15,16]. However, these methods focus only on the efficiency of energy consumption, which is problematic in that it causes degradation of the recognition rate. The activity recognition engine has a decision cycle of a few seconds, whereas human behavior has a much longer cycle. Therefore, analyzing all sequence data for the same activity over a long period with the activity recognition engine results in wasted energy. Optimization of the entire HAR system would therefore require a method for recognizing human behavioral change in the initial segmentation procedure.

In this paper, we proposed an energy-efficient method for human activity recognition with segment-level change detection and deep learning. CNNs are currently widely used in inertial-sensor-based HAR and are known for their high computational complexity and energy consumption. The energy consumption of the CNN is closely related to the network architecture, especially the number of network parameters. Because the CNN parameters occupy most of the fully connected layers, the proposed method effectively reduces the number of network parameters by using a fully convolutional network (FCN) in which the fully connected layers are replaced by convolutional layers. Furthermore, considering that human activities such as walking, jogging, and running have a longer cycle than HAR methods, which have cycles of a few seconds, it is possible to reduce the amount of computation by detecting the time at which the activity changes and by recognizing the activity only at that time. Therefore, we propose segment-level change detection, which enables the identification of activity change with very low computational complexity compared to the activity classification engine.

In summary, this research makes the following contributions. First, we propose a method that can detect activity change by monitoring the compact features of IMU signals. Second, we propose an efficient activity recognition method based on FCN and devise an energy-efficient activity recognition method that detects activity change and recognizes the activity only when necessary. Finally, we conducted experiments using a public dataset to demonstrate that the proposed method can effectively reduce energy consumption without sacrificing accuracy.

The remainder of this paper is organized as follows. Section 2 briefly reviews related work on HAR approaches for efficient energy consumption. Section 3 outlines the proposed energy-efficient HAR method. Section 4 discusses the various experiments conducted and the results obtained. Finally, Section 5 concludes this paper.

## 2. Related Work

Deep learning has lately been used successfully in the fields of computer vision and speech recognition. This has encouraged many researchers to use deep learning with inertial sensor data for classifying human activities. Several methods using neural networks such as CNNs [4,5,6,7,8], RNNs [9,10,11], and RBMs [1] have been proposed to recognize human activities. Among these methods, CNNs have been widely used because they are robust to changes in motion intensity and are able to capture the local dependencies of a sensor signal [4].

Zeng et al. [4] introduced an approach based on CNN to extract meaningful features without domain knowledge to classify human activities. Their proposed CNN comprises one pair of layers, convolutional and pooling layers, and two fully connected layers. The experimental results show that the CNN-based approach outperforms the handcrafted feature-based methods. Ronao and Cho [5] proposed CNN for classifying activities using the time-series data of the accelerometer and gyroscope sensors and then analyzed the performance according to the network architecture and hyperparameters. Their experimental results showed that increasing the depth of the network improves the recognition rate. Jiang and Yin [6] proposed a method that generates activity images by applying Discrete Fourier Transform (DFT) to the signal image, which is achieved by stacking the original sensor signals. They used the signals of the gyroscope, total acceleration, and linear acceleration to generate activity images, which they used as input into a deep CNN (DCNN) to classify human activities. They used a support vector machine (SVM) to process the DCNN results with a low probability to improve the performance of activity recognition. Rueda et al. [7] proposed architecture referred to as a CNN-IMU network for recognizing activities in an order-picking scenario. The architecture comprises parallel branches to process the multi-channel time-series data acquired from multiple IMUs and each branch employs temporal convolution and max-pooling operations to each type of sensor data. The branches create an intermediate representation of the raw sensor signals, after which the results of each branch are concatenated to those of the fully connected layers to classify the activities. Hur et al. [8] proposed an efficient encoding scheme to generate a precise image of the sensor signals and then they classified the activities by using image-based CNN models. Unlike conventional methods, which only use the integer part of sensor signals, they separated the sensor signals into three parts: the integer, the first two decimal places, and the next two decimals, and map each part to the color channel of the image to minimize the distortion. The image-based CNN model comprises six convolutional layers, two max-pooling layers, a fully connected layer, and a softmax layer. Cho and Yoon proposed an activity recognition method that employs a two-stage activity recognition process and test data sharpening [17]. They first classified the dynamic and static activities and then recognized the individual activities using a 1D CNN model. More specifically, they used a binary 1D CNN model for the first stage and two 3-class 1D CNN models for the second stage. Data sharpening, which emphasizes its high-frequency components, is used to improve the activity recognition accuracy.

The previous work mainly focused on increasing the recognition rate by designing the network architecture or by devising a precise encoding scheme for network input. This is because increasing the recognition rate is the primary goal in the field of HAR. However, recent research [18] pointed out that the energy consumption between traditional handcrafted approaches and that of deep neural networks differs significantly. Therefore, researchers have recently begun to recognize the necessity for methods that are both accurate and energy efficient.

Several energy-efficient approaches for human activity recognition have been proposed. Energy-efficient approaches based on handcrafted features usually adjust the sampling rate [12,13] or use lightweight features to reduce energy consumption [19,20]. However, deep neural networks typically cannot flexibly process changes in the size of input data. Furthermore, sensor signals with a low sampling rate would make it difficult for DNNs to learn robust features from the limited information. Therefore, previous methods for saving energy consumption cannot be applied to activity recognition methods based on DNNs.

Few studies aimed at the implementation of energy-efficient activity recognition based on DNNs have been reported [14,15,16]. Ravi et al. [14] proposed a resource efficient method for identifying human activities on low-power devices. They reduced the complexity of the neural networks by using a shallow network that comprises a temporal convolutional layer, a fully connected layer, and a softmax layer. A spectrogram of a sensor signal represents the signal as a function of frequency and time and it was used as input into the shallow network to consider the variation of sensor configuration. Although they demonstrated that the proposed method can recognize human activity in real-time on low-power devices, the accuracy of their method was lower than that of the method based on handcrafted features. To increase the recognition rate of human activities, Ravi et al. [15] also proposed a neural network that combined handcrafted features and the features extracted from a shallow network [14]. Although they fused features with different properties, the accuracy of their methods was similar to those using handcrafted features only. Their method using a shallow neural network was proposed to reduce the computational complexity, but it is problematic because of the degradation of the activity recognition rate. An activity recognition method using FCNs was proposed to take into account the tradeoff between the recognition rate and memory usage [16]. This method used DNNs comprising five convolutional layers to reduce the number of parameters and improved the recognition rate compared to the method using shallow neural networks. As mentioned above, a few research efforts have been devoted to the optimization of the classification engine of the HAR system for energy efficiency; however, the optimization of the entire HAR system including the segmentation process has not been studied.

## 3. Energy-Efficient Human Activity Recognition Method

**Figure 1:**
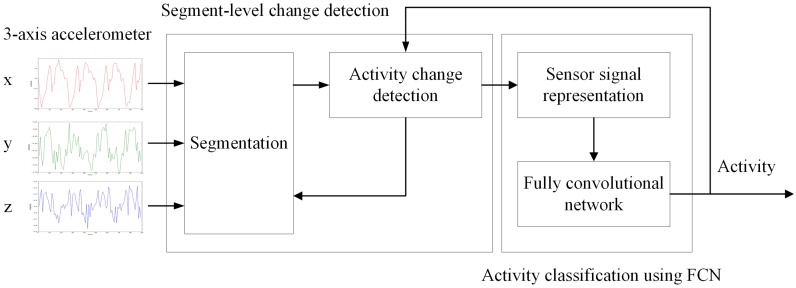
Proposed energy-efficient human activity recognition method.

In this section, we elaborate on our energy-efficient human activity recognition method. A flowchart of the proposed method is shown in Figure 1. The method performs two tasks: segment-level change detection and activity classification using FCN. First, segmentation is performed on 3-axis accelerometer signals and, a lightweight feature is extracted from a segment to compare the difference between the activity of the current and the previous segment. If the change in feature values is smaller than the threshold, the activity of the current segment is considered the same as the previous activity. If the change in feature values exceeds the threshold, the current segment of the sensor signal is examined more precisely by using FCN. In this step, sensor signals are encoded to a 2D image and used as input into FCN to recognize the activity. Leveraging segment-level change detection allows the frequency of FCN usage to be decreased; consequently, it becomes possible to reduce the high computational costs associated with this process.

### 3.1. Segment-Level Change Detection

The first step of a typical activity recognition process entails collecting raw signals from the inertial sensors. This study only considers accelerometers but the results can be extended to time-series data from other sensors such as gyroscopes, magnetometers, and electrocardiography sensors. The total acceleration signals collected from the accelerometer are separated into body acceleration and gravity acceleration components. The proposed method uses the total acceleration data for segment-level change detection and the set of total acceleration and body acceleration data for activity classification using FCN.

Because the raw signal from accelerometers is collected continuously, segmentation is performed on these raw signals, which are divided into minimum units for activity recognition. The sliding window as a segmentation approach is widely used for activity classification and it is divided into a Fixed-size Non-overlapping Sliding Window (FNSW) and a Fixed-size Overlapping Sliding Window (FOSW) depending on whether the windows overlap. The proposed method uses the FOSW approach because the HAR method based on this approach is known to produce superior results [21].

Segment-level activity change detection is achieved by using Signal Magnitude Area (SMA) [22] as a feature. SMA is used to measure the magnitude of a varying quantity and is utilized to distinguish between signals associated with the activity and the remaining signals [23]. As SMA can measure the level of physical activity from accelerometer signals [24], we monitor the value of SMA to identify the activity changes.

SMA can take both the effects of the magnitude and duration of the accelerometer signal into consideration, which calculates the summation under the magnitude of acceleration over three axes of each window normalized by the window length. The discrete form of the SMA can be calculated as follows [25]

(1) $$SMA=\frac{1}{w}\left(\sum_{i=1}^{w}\left|x_{i}\right|+\sum_{i=1}^{w}\left|y_{i}\right|+\sum_{i=1}^{w}\left|z_{i}\right|\right),$$

where *w* is the window size, and $x_{i}$, $y_{i}$, and $z_{i}$ are the *i* th samples of the sensor signal of the *x*-, *y*-, and *z*-axis in a window, respectively.

The following equation is used to determine whether the activity of a current window changes by calculating the difference between the SMA value of the current window and that of the previous window:

(2) $$W\left(t\right)=\{\begin{array}{cc} \mathrm{new}\;\mathrm{activity}, & \mathrm{if}\left|SMA(t)-SMA(t-1)\right|\geq T\\ \mathrm{same}\;\mathrm{activity}, & \mathrm{if}\left|SMA(t)-SMA(t-1)\right|<T \end{array},$$

where W(t) is the current window, *T* is the threshold for determining the activity change, and SMA(t) and SMA(t−1) is the SMA value of the time *t* and t−1, respectively.

Generally, the activity recognition method classifies the activity by using data that are continuously acquired by the IMU sensor worn by a specific user and the proposed method identifies activity change by comparing the difference between the SMA values of the current activity and those of the previous activity. Thus, the proposed system is independent from the subjects. However, similar activities such as walking and ascending stairs can produce highly similar SMA values. Therefore, we perform an activity classification using the FCN model every *N*-th frame when no change in behavior occurs. Our expectation is that performing activity classification using FCN at specific intervals would be able to decrease the error caused by the similarity of SMA values of different activities.

**Figure 2:**
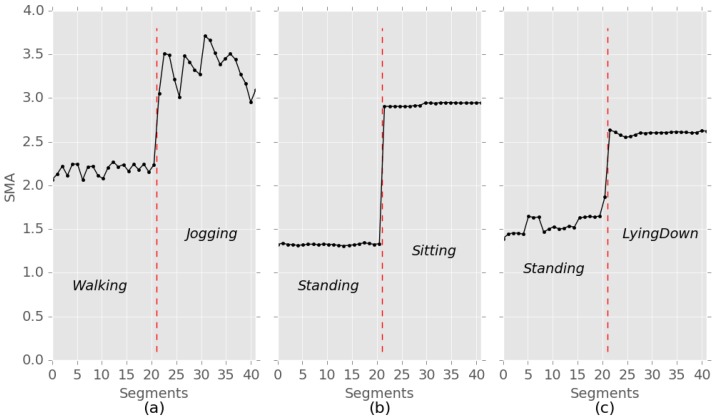
SMA according to the activity changes of three subjects. Activity transition from (**a**) walking to jogging, (**b**) standing to sitting, and (**c**) standing to lying down.

The SMA calculated for each segment of accelerometer signals is shown to illustrate activity transitions in Figure 2. These results show that the inter-activity variation of SMA is greater than its intra-activity variation. Rather than achieving accurate activity classification, the goal of change detection using SMA is to capture the moment at which activity change occurs. Therefore, SMA would be a good choice to identify changes in activities.

**Figure 3:**
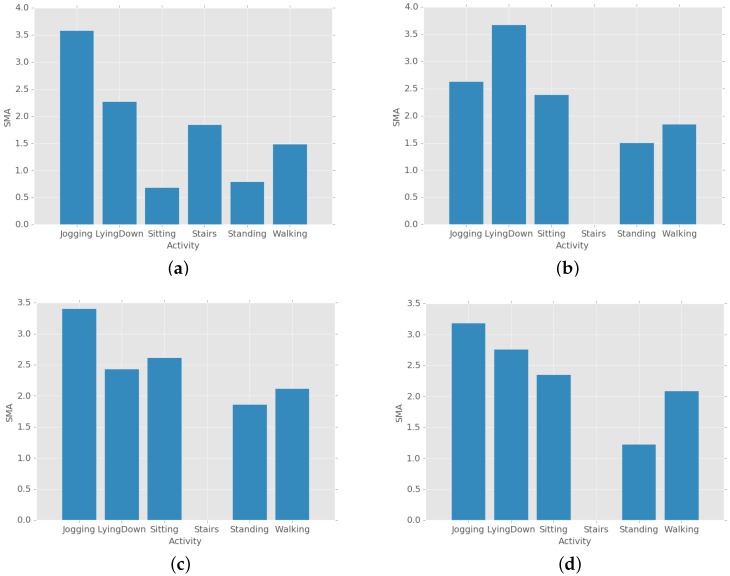
Examples of the average SMA values of the activities of four subjects (**a**) subject id 194 (**b**) subject id 650 (**c**) subject id 565 (**d**) subject id 585.

Figure 3 shows examples of the average SMA values of the activities of four subjects calculated using a public dataset [26]. The dataset was constructed by using smartphones to collect signals in uncontrolled environments and were gathered from 563 individuals. Figure 3 indicates that the SMA values are a good measure to distinguish the different activities even though the SMA values of the same activity can vary among subjects.

### 3.2. Activity Classification Using FCN

Lately, several methods using various neural networks such as CNNs [4,5,6,7,8], RNNs [9,10,11], and RBMs [1] have been proposed to recognize human activity and their performance has proven to be successful. Especially, CNNs are a popular choice for classifying activities because they consider the local dependencies of the input signal and are robust to changes in the magnitude of the motion [4]. Previous work using CNNs mainly focused on increasing the recognition rate; thus, deeper and more complex network architectures were devised. However, methods for classifying human activities would need to consider energy consumption to ensure that they are suitable for deployment in real-world applications.

The energy consumption of CNNs is closely related to their network architecture, especially the depth of the network and the number of network parameters. The “Network-in-Network” (NiN) architecture was recently proposed together with global average pooling (GAP), which replaces the fully connected layers in CNN [27]. GAP can represent the relationship between feature maps and categories more effectively than a fully connected layer and is more robust to spatial translations of the input data [27]. In addition, our previous work [16] proved that FCNs using GAP can reduce the number of network parameters without sacrificing the recognition rate. Therefore, we used FCNs to reduce the number of network parameters and varied the network depth to find the optimal network structure.

The proposed method transforms the sensor signals into 2D images to use as input for the FCN for activity recognition. The total acceleration and body acceleration of a 3-axis accelerometer are used to generate a 2D image. The body acceleration signal was obtained by applying a third-order high-pass Butterworth filter with a cutoff frequency of 0.3 Hz. The magnitude of the total acceleration and body acceleration were also used because the magnitude of the accelerometer signal is a significant metric for distinguishing activities [28]. Given eight signal sequences, each signal sequence is stacked as a row of a matrix and is then transformed to a predefined image size. The generated image is used as input into the FCN to classify the activities.

**Figure 4:**
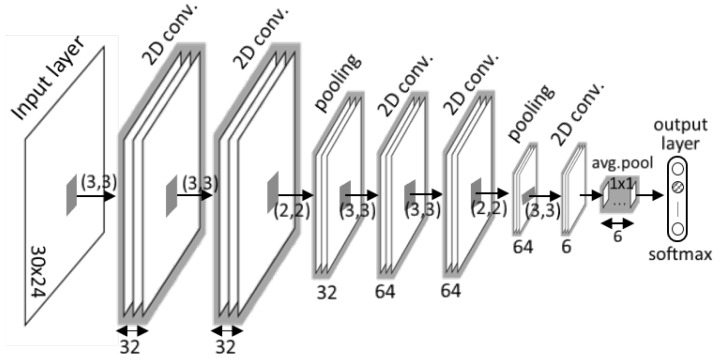
Example architecture of fully convolutional networks for classifying activities (FCN-III).

In this study, four FCN models with various shapes were considered to find the best network structure for identifying activities. Each network model has a different number of convolutional layers and every convolutional layer uses a rectified linear unit (ReLU) as the activation function. GAP is used to replace the fully connected layers. Details of the FCN models are summarized in Table 1. For example, the architecture of FCN-III comprises five convolutional layers, two max pooling layers, and one global average-pooling layer as illustrated in Figure 4.

## 4. Experiments

Existing methods focus on increasing the activity recognition rate; hence, datasets that are pre-processed and segmented by activities are popularly used for experimentation. However, to evaluate the proposed method, we need raw sensor signals collected sequentially with a timestamp that reflect various activities in sequence. Therefore, we evaluated our method using the WISDM v2.0 dataset [26,28], which is publicly available. This dataset was constructed by using smartphones to collect signals in uncontrolled environments and comprises 2,980,765 samples representing six activity classes, i.e., walking, jogging, ascending and descending stairs, sitting, standing, and lying down, from 563 individuals [26,28].

We first evaluate the activity recognition rate of the proposed FCN models and compare the result with the recognition rate of a conventional CNN model. Then, we explore the effectiveness of the proposed method which adopts segment-level change detection. Finally, we compare the energy consumption of the proposed method with that of the conventional CNN model on embedded platforms.

### 4.1. Activity Recognition Performance

We investigated the activity recognition rate of the proposed FCN models using the WISDM dataset. We applied 10-fold cross-validation to evaluate the generalization performance of the proposed method. The dataset was randomly partitioned into 10 equally sized subgroups. Of these 10 groups, a single group was used as the test set, and the remaining nine groups were used as the training set. The cross-validation procedure was repeated 10 times, with each of the 10 subgroups used exactly once as the test data. The dataset was collected with a sampling rate of 20 Hz and the proposed method segmented the raw signals by using a fixed-size window of 4.5 *s* (90 samples) with 50% overlap. As a result, the dataset produced a total of 65,067 segmented data values. For each window, the body acceleration and its magnitude were calculated and then stacked row-by-row along with the total acceleration and its magnitude. The stacked signals were transformed into a 30 × 24 image and then used as input into the FCN model.

All FCN models used the adaptive moment estimation (ADAM) optimizer [29] with a constant learning rate of 0.0001 and the batch size was set to 32. Dropout with a probability of 0.25 was applied before the last convolutional layer. The number of epochs was set to 400 and an early termination method was used to prevent over-fitting. In the training step, we used 10% of the training set as validation data. Our experiments were conducted using Python with the Keras library using TensorFlow as backend.

**Figure 5:**
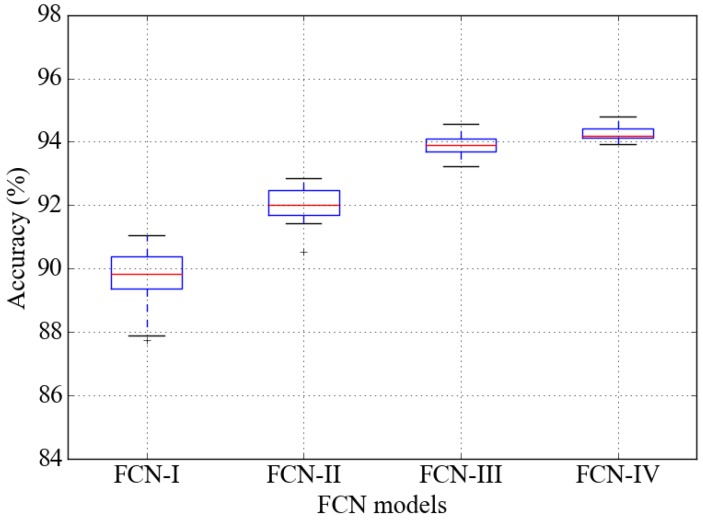
Performance comparison of the FCN models.

The performance of the FCN models is compared in Figure 5. We measured the performance by applying 10-fold cross-validation and the mean accuracy is indicated by the red rectangles in the figure. The experimental results show that the deeper networks achieve a higher activity recognition rate. We adopted the FCN-III model as the activity recognition engine of our proposed method because it showed similar performance to the FCN-IV model despite having fewer network parameters.

In addition, we compared the performance of the proposed FCN model with that of the CNN model. The CNN model has a network structure similar to that of the FCN-III model but the last convolutional layer and the GAP layer of the FCN model are replaced by two fully connected layers comprising 256 neurons. The ReLU activation function and dropout with a probability of 0.5 were applied to the two fully connected layers. We also compared the accuracy of the proposed activity recognition method with that of recent methods [15,30]. Table 2 compares the performance of the proposed FCN model with that of the CNN model and those of recent methods. The accuracy of activity recognition depends on the parameters of segmentation methods such as the length of a frame and the sampling strategy. Despite using a shorter frame length, the recognition rate of the proposed models is higher than that of the recent methods. In Table 2, the recognition rate of the CNN model is slightly higher than that of the proposed FCN model. However, considering the huge difference in the number of network parameters, this result confirms the recognition rate of the proposed FCN model to be excellent in terms of computational efficiency and power consumption.

### 4.2. Performance of the Human Activity Recognition Method

In this section, we explore the effectiveness of adopting segment-level change detection for the proposed method. To test the performance of the overall activity recognition system, we used all the data of the WISDM dataset as test data because continuously collected data is necessary to evaluate the performance of the proposed method to best mimic uncontrolled real-life environments. The proposed method segmented the raw signals by using a fixed-size window of 4.5 *s* (90 samples) and 50% overlap and the SMA was calculated using the data contained in half of the window. If the change in the SMA values was smaller than the threshold, the activity of the current window was considered the same as the previous activity. On the other hand, if the SMA difference between the current window and the previous windows exceeded the threshold, the proposed method called the FCN model to classify the activities. The threshold for determining the activity change was manually defined. In addition, in the absence of any change in behavior, the proposed method used the classifier to perform activity classification every 30 frames.

**Figure 6:**
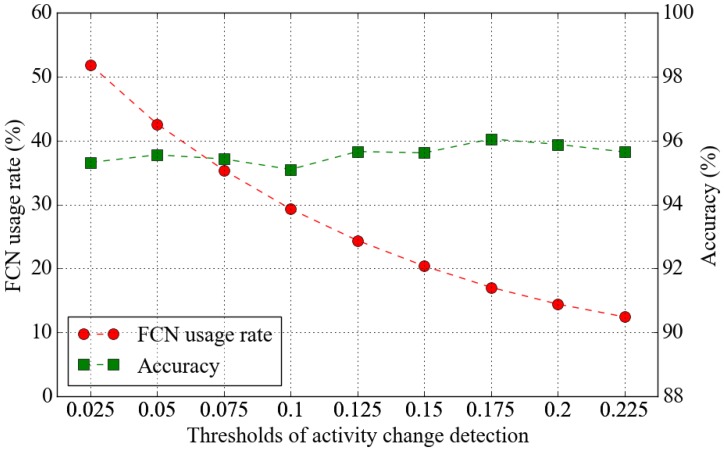
FCN usage rate and accuracy of activity recognition according to the thresholds of activity change.

To find the optimal threshold for determining the activity change, we conducted the experiment with various threshold values. Among the models generated by the result of 10-fold cross-validation, we used the FCN model closest to the average recognition rate of cross-validation. We measured the accuracy of activity classification with respect to the threshold values. The experimental results, which are plotted in Figure 6, show that the adoption of segment-level change detection by the proposed method retains the accuracy of activity recognition even when the frequency of use of the FCN model is reduced by 90%. This is because the human behavior change cycle is much longer than the analysis cycle of the activity recognition engine. The overall energy consumption of the activity recognition system is expected to be reduced by reducing the frequency of use of the FCN model, which is computationally expensive. As a result of this experiment, we set the thresholds for activity change detection in the remaining experiments to 0.2.

**Figure 7:**
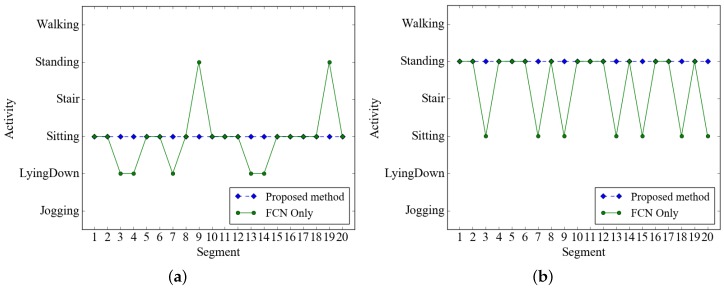
Activity change detection is effective for classifying a series of identical activities. The FCN model occasionally classifies (**a**) sitting activity as standing or lying down (**b**) standing activity as sitting.

Exploration of the effectiveness of segment-level change detection requires continuously collected data that contain the activity change. However, because the dataset used for measuring the performance of activity classification is partitioned without considering the order of the data, we used all the data of the WISDM dataset containing the recordings of 563 individuals. We compared the recognition rates of activity classification methods according to whether classifiers and segment level change detection are applied. We used the FCN and CNN model as classifiers and selected the models closest to the average recognition rate of cross-validation, respectively. The activity classification results of the proposed method are provided in Table 3. In Table 3, “Accuracy for test data” means the test result of 10-fold cross-validation. The experimental results show that the recognition rate of the proposed method is retained when activity change is adopted. The ratio classified by activity change detection was 86% and this means that classifying human activities continuously in a short cycle is highly inefficient; moreover, activity change detection using compact features with low computational complexity can largely replace the use of a classification algorithm based on deep learning to reduce the computational complexity and energy consumption. In addition, activity change detection can reduce the number of false positives classified by the FCN model because it has a smoothening effect on the activity recognition results, as shown in Figure 7. This figure shows that continuous activity is sometimes misclassified by FCN models, but the proposed method ensures continuity of action.

### 4.3. Energy Consumption on Embedded Platforms

**Figure 8:**
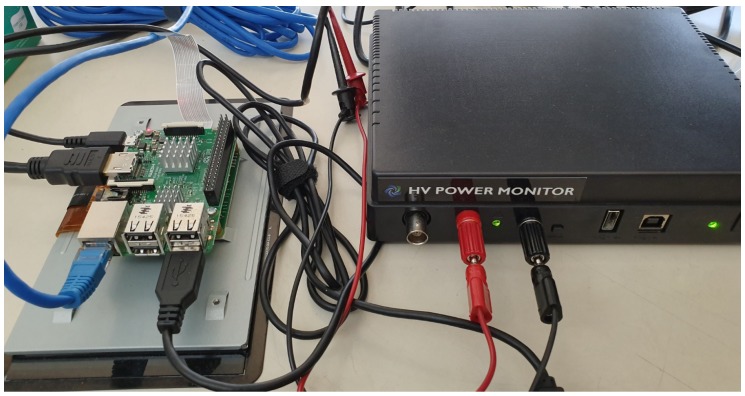
Experimental environment for measuring power consumption on embedded platforms.

A user activity must be monitored over a long period of time, which is inefficient in terms of energy consumption. However, the need to frequently recharge monitoring devices can result in user dissatisfaction. Thus, energy consumption needs to be taken into account when methods using deep learning are deployed in embedded devices. This led us to explore the energy consumption of the activity recognition method based on deep learning on embedded platforms. We used two embedded platforms, namely Raspberry Pi 3 with a 1.4 GHz 64-bit quad-core processor and 1 GB RAM, and the Jetson Nano with a 1.4 GHz quad-core 64-bit CPU, a 128-core integrated NVIDIA GPU, and 4 GB RAM. The Jetson Nano has two power modes, i.e., a 5-Watt mode for supporting limited power and a 10-Watt mode for maximizing the performance. The energy consumption of the activity recognition methods running on embedded platforms was measured using the Monsoon power monitor tool. The experimental setup for measuring energy consumption is shown in Figure 8.

The energy consumption of the activity recognition methods is calculated by measuring the amount of energy used to process all of the segmented data in the WISDM dataset. Therefore, we consider all subjects in the WISDM dataset. The proposed system emulates the sensor by using the public dataset because the proposed system does not gather data from the IMU sensor directly. To mimic the sensor, the proposed system reads the sensor data from file and then, feeds the sensor data to the activity classification method. Thus, we first measured the energy consumption by the operating system and the energy consumption by the process emulating the IMU sensor. We refer to this energy consumption as the baseline energy consumption (baseline). Subsequently, we compared the energy consumption of the FCN-based method with that of the CNN-based method by excluding the baseline energy consumption. Finally, we measured the energy consumption by the CNN-based and FCN-based methods with segment-level change detection. Throughout this section, we refer to the CNN-based method with segment-level change detection as CNN-S and to the FCN-based method with segment-level change detection as FCN-S. We also measured the processing time of the activity recognition methods on the embedded systems.

**Figure 9:**
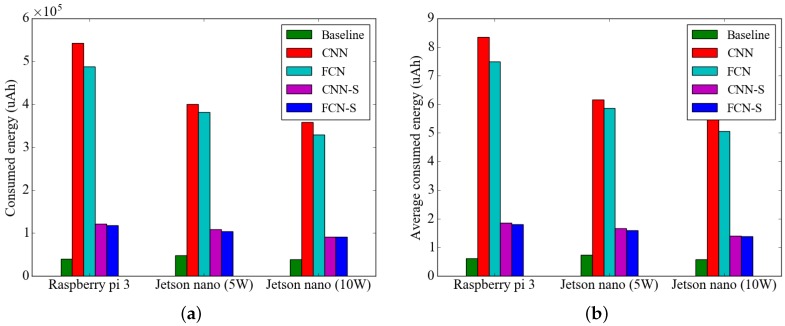
Results of measurements of energy consumed by the activity recognition methods on the embedded platforms: (**a**) Total energy consumed (**b**) Average energy consumed per sample.

The results of the energy consumption measurements of the activity recognition methods on the embedded platforms are shown in Figure 9. Total consumed energy refers to the energy required to process all 65,067 segmented data values in the WISDM dataset. In Figure 9a, the experimental results show that the total amount of energy consumed by the activity recognition method using CNN is more than 4.6 times higher than that of the FCN-based method with segment-level change detection. Compared with the CNN-based method and FCN-based method without segment-level change detection, the FCN-based method reduces the energy consumption by approximately 10% compared to the CNN-based method. Moreover, the experimental results show that segment-level change detection can reduce the energy consumption of the FCN-based and CNN-based methods by approximately 84%. The average energy consumption per sample of the activity recognition methods is presented in Figure 9b and the experimental results show that the proposed method consumes 1.39 μAh of energy to analyze one sample with a duration of 2.25 s. The proposed method consumed 5.3% of the energy of a device with 1000 mAh battery capacity to analyze the data collected by the sensor in one day, suggesting that it could be used for 19 days without recharging.

**Figure 10:**
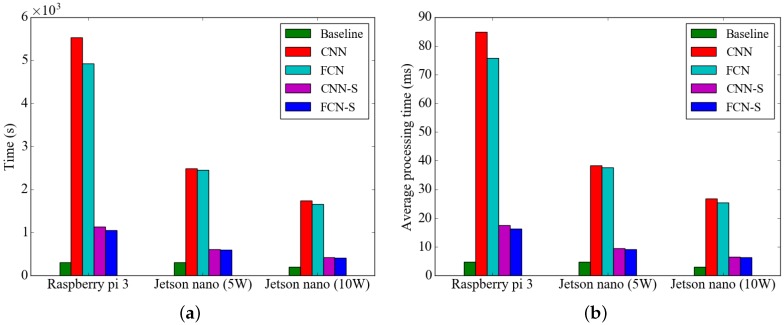
Processing time of activity recognition methods on embedded platforms (**a**) Total processing time (**b**) Average processing time per sample.

The processing time of the activity recognition methods on the embedded platforms is shown in Figure 10. The total processing time of the activity recognition methods is the time required to process all 65,067 of the segmented data points in the WISDM dataset. We measured the processing time of IMU sensor emulation, which reads the sensor data from the file and feeds the sensor data to the activity classification method. We refer to this processing time as the baseline processing time (baseline). As shown in Figure 10a, the activity recognition method using the CNN model requires a minimum of four times more processing time than the proposed method. Figure 10b shows that the proposed method can process sample data collected for a period of 2.25 s on a high-end embedded platform in an average of 6.3 ms and requires an average of 16 ms on a low-end embedded platform. The experimental results confirmed that we could reduce the energy usage significantly while maintaining the recognition rate of human activity by optimizing the entire HAR system as a result of implementing segment-level change and FCN.

## 5. Conclusions

In this paper, we proposed an energy-efficient method for the overall system of human activity recognition. Unlike many existing methods that focus on reducing the energy consumption of the activity classification engine, our approach involved optimizing the segmentation process. Because human activities have a longer cycle than HAR methods, which perform their analysis in cycles of a few seconds, classifying human activities continuously by using a short cycle is highly inefficient. Therefore, we proposed the segment-level change detection method to identify activity change with extremely low computational complexity. Subsequently, an FCN model, which has a high recognition rate, was applied to classify the activity only when activity change occurs. We compared the recognition rate of the proposed method with that of the CNN-based method using a public dataset and explored the energy consumption of the activity recognition methods that use deep learning on embedded platforms. The experimental results showed that the proposed FCN model achieves a recognition rate similar to that of the CNN model despite using only 10% of the network parameters of the CNN models. We also verified that the adoption of segment-level change detection in the proposed method retains the accuracy of activity recognition even though the frequency of use of the FCN model is reduced by 90%. The experiments that were conducted to measure the energy consumption on the embedded platforms showed that the proposed method uses as much as 6.5 times less energy than the CNN-based method when comparing only the energy consumed for activity recognition, excluding the power essential for system operation. The proposed method consumes 1.39 μAh of energy to analyze one sample of 2.25 s long, hence it consumed only 5.3% of the energy of a device with 1000 mAh battery capacity to analyze the sensor data collected in a day. The experimental results proved that the proposed method offers a suitable and practical solution to reduce the energy consumption of a human activity recognition system without sacrificing the recognition rate of human activity classification.

This paper presented the experimental results for embedded devices by using a dataset constructed in advance, but it would be necessary to verify the performance by using a smartphone to capture signals in actual everyday life. Thus, in the future, we plan to deploy the proposed method on a smartphone to evaluate the performance of our method in real environments.

## Figures and Tables

**Table 1:** Network parameters.

Layer	FCN-I	FCN-II	FCN-III	FCN-IV
1	Input (30 × 24)	Input (30 × 24)	Input (30 × 24)	Input (30 × 24)
2	Conv. 16@(3 × 3)	Conv. 32@(3 × 3)	Conv. 32@(3 × 3)	Conv. 32@(3 × 3)
3	Conv. 16@(3 × 3)	Conv. 32@(3 × 3)	Conv. 32@(3 × 3)	Conv. 32@(3 × 3)
4	Max Pooling (2 × 2)	Max Pooling (2 × 2)	Max Pooling (2 × 2)	Conv. 32@(3 × 3)
5	Conv. 32@(3 × 3)	Conv. 64@(3 × 3)	Conv. 64@(3 × 3)	Max Pooling (2 × 2)
6	Max Pooling (2 × 2)	Max Pooling (2 × 2)	Conv. 64@(3 × 3)	Conv. 64@(3 × 3)
7	Conv. 6@(3 × 3)	Conv. 6@(3 × 3)	Max Pooling (2 × 2)	Conv. 64@(3 × 3)
8	GAP	GAP	Conv. 6@(3 × 3)	Max Pooling (2 × 2)
9	Softmax	Softmax	GAP	Conv. 6@(3 × 3)
10			Softmax	GAP
11				Softmax

**Table 2:** Results of activity classification.

Network Model	Accuracy (%)	Number of Parameters	Segmentation
FCN model	93.89 ± 0.37	71,184	4.5 *s* segment, FOSW
CNN model	94.64 ± 0.28	820,710	4.5 *s* segment, FOSW
Catal et al. [30]	89.8	-	10 *s* segment, FNSW
Ravi et al. [15]	92.7	-	10 *s* segment, FNSW

**Table 3:** Results of activity classification (the threshold for activity change detection was set to 0.2).

	FCN Model	CNN Model
Accuracy for test data	94.06%	94.66%
Accuracy for all data w/o activity change detection	95.63%	97.36%
Accuracy for all data with activity change detection	95.89%	97.28%
FCN usage rate	14.44%	14.44%

## Abbreviations

The following abbreviations are used in this manuscript:

ADAM	ADAptive Moment estimation
CNN	Convolutional Neural Networks
DFT	Discrete Fourier Transform
DNN	Deep Neural Networks
FCN	Fully Convolutional Network
FNSW	Fixed-size Non-overlapping Sliding Window
FOSW	Fixed-size Overlapping Sliding Window
GAP	Global Average Pooling
HAR	Human Activity Recognition
IMU	Inertial Measurement Unit
RBM	Restricted Boltzmann Machines
RNN	Recurrent Neural Networks
SMA	Signal Magnitude Area
SVM	Support Vector Machines
